# PA‐MSHA inhibits the growth of doxorubicin‐resistant MCF‐7/ADR human breast cancer cells by downregulating Nrf2/p62

**DOI:** 10.1002/cam4.938

**Published:** 2016-10-18

**Authors:** Yingze Wei, Danyang Liu, Xiaoxia Jin, Pan Gao, Qingying Wang, Jiawen Zhang, Nong Zhang

**Affiliations:** ^1^ Department of PathologySchool of Basic Medical SciencesFudan UniversityShanghaiChina; ^2^ Department of PathologyNantong Tumor HospitalNantongJiangsuChina; ^3^ Department of Obstetrics and GynecologyShanghai Tenth People's HospitalTongji UniversityShanghaiChina

**Keywords:** Breast cancer, doxorubicin resistance, Nrf2, p62, PA‐MSHA

## Abstract

Acquired resistance to doxorubicin in breast cancer is a serious therapeutic problem. In this study, we investigated whether *Pseudomonas aeruginosa* mannose‐sensitive hemagglutinin (PA‐MSHA) could inhibit the growth of doxorubicin‐resistant breast cancer cells. We found that the expressions of Nrf2 and p62 in breast cancer were higher than that in the corresponding adjacent normal tissues and benign breast epithelial cell. The expressions of Nrf2 and p62 in breast cancer doxorubicin‐resistant cells MCF‐7/ADR were higher than that in doxorubicin‐sensitive cells MCF‐7. Silencing of Nrf2 or p62 rendered breast cancer cells more susceptible to doxorubicin. We further demonstrated that PA‐MSHA inhibited growth and induced apoptosis of MCF‐7/ADR cells but not MCF‐7 cells. Subcutaneous administration of PA‐MSHA greatly inhibited the growth of xenograft tumors from MCF‐7/ADR cells in nude mice. In addition, PA‐MSHA could downregulate Nrf2 and p62 in vitro and in vivo. These results suggested that activation of Nrf2 and p62 was associated with doxorubicin resistance in breast cancer. PA‐MSHA could inhibit the growth of doxorubicin‐resistant MCF‐7/ADR cells and its potential mechanism might be due to the suppression of Nrf2/p62. It indicated the possibility of using PA‐MSHA in doxorubicin‐resistant breast cancer.

## Introduction

Breast cancer is the most common cause of cancer‐associated mortality among females worldwide. In China, it is the most frequently diagnosed malignant tumor and the sixth cause of cancer death in females, with an estimated 248,620 new cases and 60,473 deaths in 2011 1. Therapy for primary breast cancer usually involves surgery combined with radiotherapy, endocrine therapy, and/or chemotherapy. Approximately 30% of patients receive chemotherapy, including postoperative and neoadjuvant chemotherapy 2. Doxorubicin (Dox) is a widely accepted chemotherapy drug used to suppress the growth and survival of human breast cancer cells. However, intrinsic and acquired resistance to doxorubicin is common in breast cancer treatment and leads to subsequent treatment failures and recurrences 3. Once drug resistance develops, higher doses of available drugs may be ineffective. Therefore, there is an urgent need to find a novel agent to conquer drug resistance in breast cancer.

Sequestosome 1 (SQSTM1, p62), an immediate early response gene, has an important function in activating antiapoptotic genes and several survival signal pathways which promote cell proliferation, migration, and differentiation 4. As a multifunctional protein, p62 was recently reported to be overexpressed in a variety of diseases including neurodegenerative diseases, insulin resistance, obesity, and various cancers 5, 6. In breast cancer, overexpression of p62 was reported to be significantly correlated with aggressive biological behavior, lymph node metastasis, and 5‐year survival rate 7, 8. In addition, dysregulated p62 was contributed to the chemoresistance 9, 10. Recently, several studies demonstrated that accumulated p62 activated nuclear factor erythroid‐2‐related factor 2 (Nrf2) expression 11, and then Nrf2 further promoted p62 upregulation through direct binding to the antioxidant response element (ARE) motif of the p62 promoter 12. It suggested a positive feedback of p62 and Nrf2. Nrf2 is a potent transcriptional activator that can recognize and bind to ARE of target gene promoters, and maintain redox balance by controlling gene transcription 13. During malignant transformation, aberrant activation of Nrf2 is observed, and activation of Nrf2 promotes cancer development and contributes to chemoresistance 14, 15. However, whether Nrf2/p62 plays an important role in breast cancer and the mechanism of resistance to Dox has remained largely unknown.

*Pseudomonas aeruginosa* mannose‐sensitive hemagglutinin (PA‐MSHA) has been reported as a new anticancer drug, which induces cell cycle arrest and apoptosis in some human cancer cells, and its role in chemotherapy is currently under investigation 16, 17. PA‐MSHA can enhance immune function of lung cancer patient and can improve chemotherapeutic effectiveness with low adverse reaction rate 18. For the malignant lymphoma patients, the clinical efficacy rate was 95.56% when they received chemotherapy plus PA‐MSHA, while it was 69.77% for the patients who received chemotherapy alone 19. Chen et al. 20 suggested that PA‐MSHA combined with TAC scheme can significantly enhance the therapeutic effect of breast cancer, lower the rate of postoperative complications, and improve the efficacy of chemotherapy. These results indicated that PA‐MSHA could play an important role in the adjuvant therapy of cancer. However, its role of chemotherapy resistance in breast cancer has not been reported so far.

In the present study, we demonstrated that Nrf2 and p62 were overexpressed in breast cancer. Nrf2 and p62 were associated with doxorubicin resistance in MCF‐7/ADR cells, and PA‐MSHA could inhibit growth of MCF‐7/ADR cells but not MCF‐7 cells by downregulating Nrf2 and p62. The objective of this study was to explore the possibility of using PA‐MSHA to conquer doxorubicin resistance and the underlying mechanisms, improving the effect of chemotherapy of human breast cancer.

## Materials and Methods

### Cell lines and reagents

Breast cancer cell lines T47D, BT549, MDA‐MB‐231, MCF‐7, and MCF‐7/ADR and benign breast epithelial cell line MCF‐10A were purchased from Chinese Type Culture Collection (Shanghai, China). MCF‐7 is doxorubicin‐sensitive cell line and MCF‐7/ADR is a human breast adenocarcinoma multidrug‐resistant cell line selected against doxorubicin. T47D, BT549, and MCF‐7/ADR cell lines were cultured in RPMI 1640 medium (Gibco, Grand Island, NY) supplemented with 10% heat‐inactivated fetal bovine serum (FBS; Gibco). MCF‐7 and MDA‐MB‐231 cell lines were cultured in Dulbecco's modified Eagle's medium (DMEM) (Gibco) supplemented with 10% heat‐inactivated FBS (FBS; Gibco). MCF‐10A cell line was cultured in a 1:1 ratio of DMEM and Ham's F‐12 nutrient mixture supplemented with 10% heat‐inactivated FBS and 1% penicillin–streptomycin, 10 *μ*g/mL insulin (Humulin 30/70), 20 ng/mL epidermal growth factor (EGF), 500 ng/mL hydrocortisone, and 100 ng/mL cholera toxin. All cultures were grown at 37°C in 5% CO_2_ atmosphere.

Doxorubicin (Dox; Adriamycin, ADR) was purchased from Sigma (St. Louis, MO). The PA‐MSHA used in the present study was purchased from Wanter Biopharma Company (Beijing, China), then scale‐diluted and stored at 4°C. Phosphate‐buffered saline (PBS 0.1 mol/L; Gibco) was used as control.

### Tissue samples

A total of 100 paraffin‐embedded, archived breast cancer samples, which were histopathologically and clinically diagnosed at the Nantong Tumor Hospital from 2006 to 2012, were used in the present study. Pathological diagnoses of breast cancer samples were managed by two experienced pathologists (S. Y. Yang and X. D. Chen) based on the World Health Organization (WHO) classification in a double‐blinded manner. The study was approved by the ethical committee of Nantong Tumor Hospital, and all patients provided written informed consent. Clinical information of the samples is summarized in Table 1.

**Table 1:** Clinicopathological characteristics of 100 breast cancer patients

Characteristics	Number of patients/Total number (%)
Age (median, range)	52.3 (32–80) years
Histological grade	
Well (I)	16/100 (16)
Moderate (II)	31/100 (31)
Poor (III)	53/100 (53)
Tumor size (cm)	
≤3.5	83/100 (83)
>3.5	17/100 (17)
Lymph node metastasis	
No	39/100 (39)
Yes	61/100 (61)

### Immunohistochemistry

The IHC procedure and the scores of Nrf2 and p62 expression were performed as reported previously 21. The sections were incubated either with rabbit anti‐Nrf2 antibody (diluted to 1:100; ab76026, Abcam, Cambridge, UK) or with mouse anti‐p62 (diluted to 1:100; sc‐28359, Santa Cruz, CA) antibody, and the omitted primary antibodies served as the negative controls. The protein levels of Nrf2 and p62 were assessed as described previously 21.

### Lentivirus transfection

Breast cancer cell lines stably silencing p62 were constructed. The negative control small hairpin RNA (shNC, 5′‐TTCTCCGAACGTGT CACGT‐3′), and p62shRNA two oligo DNAs (5′‐CGAGGAATTGACAATGGCCAT‐3′; 5′‐CCTCTGGGCATTGAAGTTGAT‐3′) were designed, synthesized, and cloned into lentivirus vectors (GV115) to form constructed GV115‐shRNA plasmids by Genechem (Shanghai, China). Breast cancer cell lines stably silencing Nrf2 were also constructed. The shNrf2 plasmid has been described previously 22.

### Western blots

Western blots were performed as described previously 23, using primary antibodies: rabbit anti‐Nrf2 antibody (diluted to 1:500; ab76026, Abcam, Cambridge, UK), mouse anti‐p62 (diluted to 1:500; sc‐28359, Santa Cruz) antibody, rabbit anti‐caspase 3 (diluted at 1:500; #9662, Cell Signaling Technology, MA), rabbit anticleaved caspase 3 (diluted at 1:500; #9661, Cell Signaling Technology), mouse anti‐Ki67 (diluted to 1:500; sc‐23900, Santa Cruz), and mouse anti‐bcl‐2 (diluted to 1:500; sc‐7382, Santa Cruz) monoclonal antibody. The mouse anti‐*β*‐actin (A1978, Sigma, MI) monoclonal antibody was diluted to 1:1000 for use as a sample loading control.

### Proliferation assay

Cells were cultured in a 96‐well plate at a density of 1 × 10^5^ cells/mL. Each well contained 1 × 10^4^ cells in a total volume of 100 *μ*L. CCK‐8 assay was performed after drug treatment for 24 h. Briefly, 10 *μ*L CCK‐8 was added in each well, and the optical density (OD) value was detected in a MULTISCAN GO‐1510 microplate reader at 450 nm (Thermo Scientific, Shanghai, China) after incubating at 37°C for 2 h. Each experimental condition was assayed in triplicate and all experiments were performed for three times.

### Morphological assay

The appearance of morphological differentiation was assessed and observed under a phase contrast inverted light microscope and photographed with a Nikon F‐601 AF Camera.

### Real‐time quantitative PCR (qPCR)

Total RNA was extracted using RNAiso Plus (Cat. #9109; Takara, Tokyo, Japan) according to the manufacturer's instructions, and then the RNA was reversed to cDNA using PrimeScript^®^ RT Master Mix (Cat. #RR036A; Takara) at 37°C for 15 min, 85°C for 5 sec, and then at 4°C. qPCR for Nrf2, p62, and GAPDH was performed in a 10‐*μ*L reaction volume using the SYBR^®^PremixEx Taq^™^ (Cat. #RR420; Takara) and ABI Prism^®^ 7900HT Real‐Time PCR System (Applied Biosystems, CA). The thermal cycle condition was one cycle at 95°C for 30 sec, followed by 40 cycles of amplification at 95°C for 5 sec, and then 60°C for 30 sec. GAPDH was used as an internal loading control. Experiments were performed in triplicate in three independent experiments.

### Apoptosis assays

The apoptosis were assayed under a fluorescence microscope following staining with Hoechst 33258 (Sigma). The cells were treated with PA‐MSHA (0.848 × 10^9^/mL) for 24 h and stained with 5 mg/L Hoechst 33258 for 30 min at 37°C, then visualized under a fluorescence microscope with standard excitation filters. Apoptotic cells were defined as cells showing nuclear and cytoplasmic shrinkage, chromatin condensation, and apoptotic bodies. Apoptotic cells were also determined using flow cytometry with an Annexin V FITC kit (Beckman Coulter, Fullerton, CA). Cells were harvested and washed with cold PBS, then resuspended with 100 *μ*L Annexin V binding buffer, and incubated with 10 *μ*L Annexin V for 15 min at room temperature in darkness. Binding buffer (400 *μ*L) containing 5 *μ*L propidium iodide (PI) was added to the cells and cells were incubated on ice for 15 min. The cells were applied to a FACS Calibur‐500 flow cytometer within 1 h of preparation. A total of 1 × 10^4^ cells were analyzed in each sample. The results were interpreted as follows: cells that were Annexin V(−)/PI(−) (lower left quadrant) were considered as living cells; Annexin V(+)/PI(−) (lower right quadrant) as apoptotic cells; Annexin V(+)/PI(+) (upper right quadrant) as necrotic cells; and Annexin V(−)/PI(+) (upper left quadrant) may be bare nuclei, cells in late necrosis, or cellular debris. Experiments were performed for three times.

### Xenograft model and treatments

Female nude mice (5–6 weeks old) were purchased from SLAC Laboratory Animal Co., Ltd. (Shanghai, China). MCF‐7/ADR cells suspensions (0.2 mL, 5 × 10^7 ^cells/mL) were injected into the left mammary fat pad of mice. When the tumor size reached 20 mm^3^, mice were randomly allocated into four groups (*n* = 5), followed by treatments: control group (subcutaneous injection PBS around tumors), doxorubicin (4 mg/kg) 24, PA‐MSHA (1.6–2.0 × 10^9 ^cells/mL), and PA‐MSHA+doxorubicin. Doxorubicin was diluted with PBS and given intraperitoneally (i.p.) once a week. PA‐MSHA (0.3 mL) was injected subcutaneously around tumors every other day. Tumor diameters were determined every 3 days with a caliper, and the tumor volume was calculated using a standard formula: tumor size (mm^3^) = width^2^ × length × 0.52. Body weights were recorded before dosing. Animal body weight was monitored every 4 days for 6 weeks. When the experiment ended, the mice were euthanized by dislocation of the cervical vertebra, and then their tumors were harvested immediately and measured.

### Histopathology

Xenograft tumor samples were collected immediately after the animals were killed and placed in 4% paraformaldehyde. Tumor sections were stained with hematoxylin and eosin for IHC. Images were captured under a light microscope at 400× magnification.

### Statistical analysis

Statistical analysis was performed using the software of Statistical Package for the Social Sciences (SPSS) Version 17.0 for Windows (SPSS, Inc., Chicago, IL). Student's *t* tests were used to determine statistical significance of differences between experimental groups. *P < *0.05 was considered significant. Graphs were created with GraphPad Prism (Version 6.01, GraphPad Software, Inc., CA).

## Results

### Expression of Nrf2 and p62 in breast cancer

**Figure 1:**
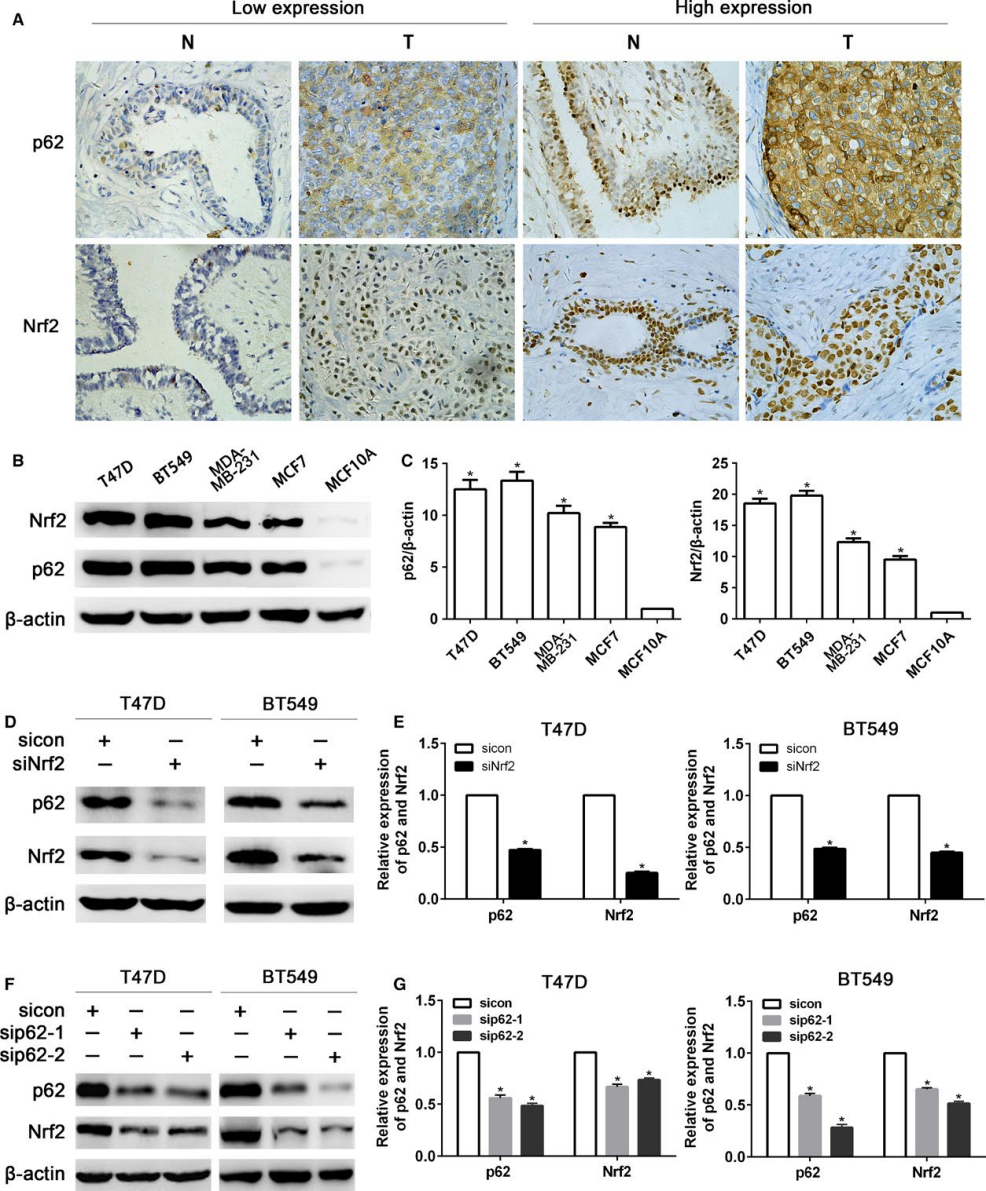
Expression of Nrf2 and p62 in breast cancer. (A) Immunohistochemical assay of Nrf2 and p62 protein levels in pairs of matched breast carcinoma tissues (T) and adjacent normal breast tissues (N). (B) Western blot analysis of Nrf2 and p62 protein levels in T47D, BT549, MDA‐MB‐231, and MCF‐7 breast cancer cell lines and MCF‐10A benign breast epithelial cell line. (C) Quantitation of Nrf2 and p62 protein levels in breast cancer cells and benign epithelial cell. (D) Western blot analysis of p62 protein level in T47D and BT549 after infecting Nrf2 shRNA lentivirus. (E) Quantitation of Nrf2 and p62 protein levels in T47D and BT549 after infecting Nrf2 shRNA lentivirus. (F) Western blot analysis of Nrf2 protein level in T47D and BT549 after infecting p62 shRNA lentivirus. (G) Quantitation of Nrf2 and p62 protein levels in T47D and BT549 after infecting p62 shRNA lentivirus. Data are presented as mean ± SD,* n* = 3, *P < 0.05.

We analyzed the protein levels of Nrf2 and p62 in 100 breast cancer tissues and corresponding adjacent normal tissues by IHC. Positive rate of Nrf2 (96/100, 96.0%) or p62 (83/100, 83.0%) was more frequently found in breast cancer tissues than in adjacent normal breast tissues (Table 2, Fig. 1A). As shown in Table 3, we noted that the protein level of Nrf2 was positively correlated with that of p62 in breast cancer tissues (*R* = 0.315, *P* = 0.013). Western blot analysis also showed overexpression of Nrf2 or p62 in T47D, BT549, MDA‐MB‐231, and MCF‐7 breast cancer cell lines in comparison to MCF‐10A benign breast epithelial cell line (Fig. 1B and C).

**Table 2:** The protein levels of Nrf2 and p62 in breast cancer tissues

Group	*n*	Nrf2		*P*	p62		*P*
		Positive *n* (%)	Negative *n* (%)		Positive *n* (%)	Negative *n* (%)	
Breast cancer	100	96 (96)	4 (4)	<0.01	83 (83)	17 (17)	<0.01
Adjacent normal breast tissues	100	40 (40)	60 (60)		1 (1)	99 (99)	

**Table 3:** The relationship between Nrf2 and p62 in breast cancer tissues

p62	Nrf2		*P*	*R*
	Positive expression	Negative expression		
Positive expression	82	1	0.013	0.315
Negative expression	14	3		

To investigate whether p62 is induced by Nrf2 activation, we introduced Nrf2 shRNA lentivirus into T47D and BT549 cells. As shown in Figure 1D and E, p62 expression was markedly reduced with Nrf2 shRNA lentivirus compared with control shRNA. Interestingly, the protein level of Nrf2 was decreased in T47D and BT549 cells treated with p62 shRNA lentivirus (Fig. 1F and G).

### The sensitivity of breast cancer to Dox determined by Nrf2/p62

**Figure 2:**
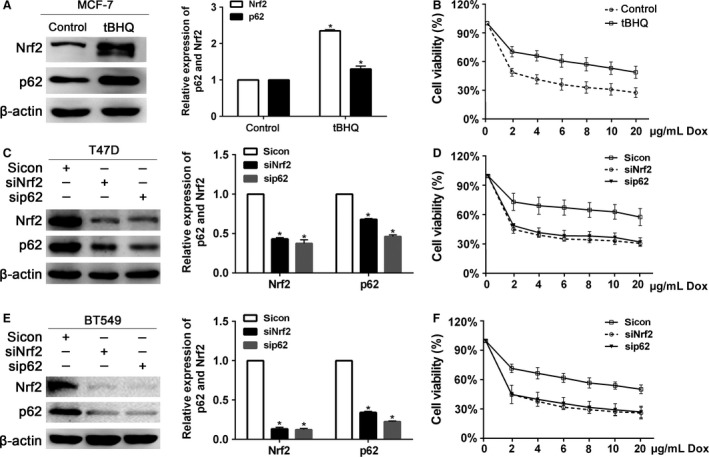
The sensitivity of breast cancer cells to doxorubicin regulated by Nrf2 and p62. (A) The protein levels of Nrf2 and p62 were determined by western blot in MCF‐7 cells after pretreated with 40 *μ*mol/L tBHQ for 24 h. (B) The tBHQ‐pretreated cells were treated with the indicated doses of doxorubicin for 24 h, followed by the CCK8 assay. (C) Protein levels of Nrf2 and p62 were determined by western blot in T47D cells infected with Nrf2 shRNA or p62 shRNA lentivirus. (D) T47D cells infected with Nrf2 shRNA or p62 shRNA lentivirus were treated with the indicated doses of doxorubicin for 24 h, followed by the CCK8 assay. (E) Protein levels of Nrf2 and p62 were determined by western blot in BT459 cells infected with Nrf2 shRNA or p62 shRNA lentivirus. (D) BT549 cells infected with Nrf2 shRNA or p62 shRNA lentivirus were treated with the indicated doses of doxorubicin for 24 h, followed by the CCK8 assay. Data are presented as mean ±SD,* n* = 3, *P < 0.05.

tBHQ (*tert*‐butylhydroquinone) is an aromatic organic compound confirmed to upregulate the protein levels of Nrf2 and p62. Pretreatment with 40 *μ*mol/L tBHQ for 24 h significantly upregulated protein levels of Nrf2 and p62 in MCF‐7 cells (Fig. 2A). In addition, the treatment with tBHQ increased the cell viability in response to Dox (Fig. 2B). As shown in Figure 2C and E, stably transfection of Nrf2 shRNA lentivirus significantly reduced the protein level of p62. Similarly, stably transfection of p62 shRNA lentivirus also reduced the protein level of Nrf2. CCK8 assay showed that transfection of Nrf2‐shRNA or p62‐shRNA sensitized T47D and BT549 cells to Dox (Fig. 2D and F).

### Nrf2 and p62 overexpressed in MCF‐7/ADR breast cancer cells

**Figure 3:**
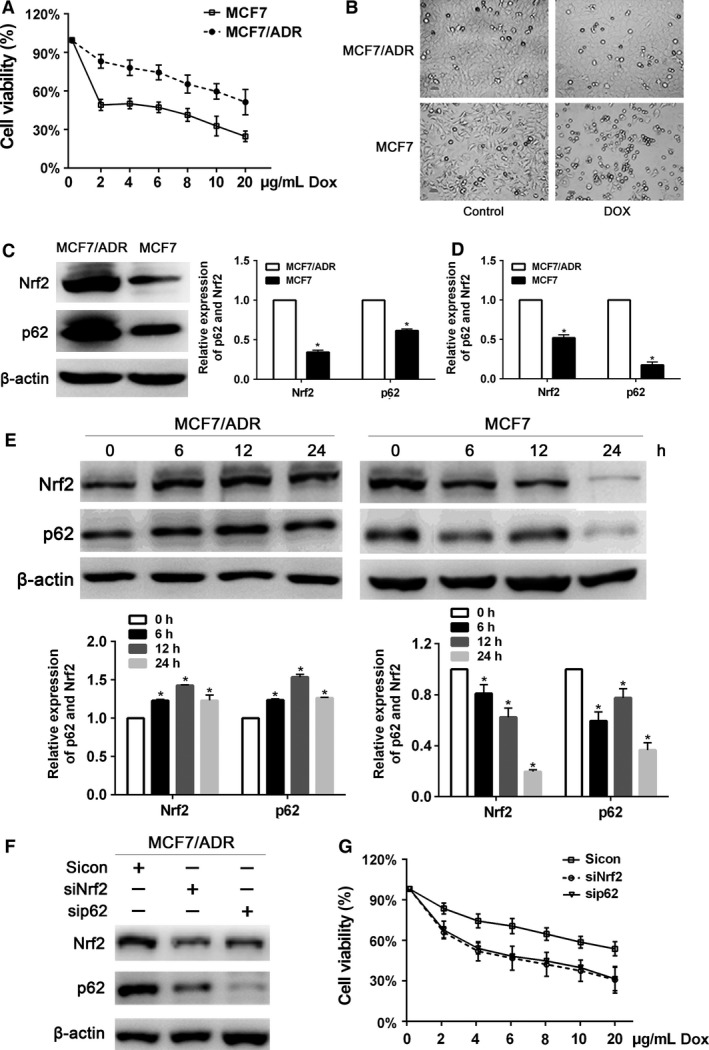
Nrf2 and p62 overexpressed in MCF‐7/ADR breast cancer cells. (A) The inhibitory effect of doxorubicin on MCF‐7 and MCF‐7/ADR cells proliferation. Cells were treated with various concentrations of doxorubicin for 24 h, and then cell viability was determined by the CCK8 assay. (B) MCF‐7 and MCF‐7/ADR cells treated with PBS or doxorubicin (3 *μ*g/mL) for 24 h were visualized by light microscopy. (C) The protein levels of Nrf2 and p62 were assessed by western blot in MCF‐7 and MCF‐7/ADR cells. (D) The mRNA expression of Nrf2 and p62 was assessed by RT‐qPCR in MCF‐7 and MCF‐7/ADR cells. (E) Western blot analysis for the protein levels of Nrf2 and p62 in MCF‐7 and MCF‐7/ADR cells treated with 3 *μ*g/mL doxorubicin. (F) MCF‐7/ADR cells were performed infected with Nrf2 shRNA or p62 shRNA lentivirus. Protein levels of Nrf2 and p62 were determined by western blot. (G) MCF‐7/ADR cells infected with Nrf2 shRNA or p62 shRNA lentivirus were treated with the indicated doses of doxorubicin for 24 h, followed by the CCK8 assay. Data are presented as mean ± SD,* n* = 3, *P < 0.05. PBS, phosphate‐buffered saline.

The percentage of MCF‐7 cell viability was decreased more significantly than MCF‐7/ADR cell when treated with different concentrations of doxorubicin for 24 h (Fig. 3A and B). As shown in Figure 3C and D, the protein level and mRNA expression of p62 and Nrf2 in MCF‐7/ADR cells were higher than those in MCF‐7 cells, respectively (*P* < 0.05). When treated with 3 *μ*g/mL doxorubicin at different times (0, 6, 12, 24 h), Nrf2 and p62 protein levels in MCF‐7/ADR cells were always upregulated, while these two proteins in MCF‐7 cells were downregulated (Fig. 3E).

In MCF‐7/ADR cells, p62 expression was markedly reduced with Nrf2 shRNA lentivirus and Nrf2 expression was decreased treated with p62 shRNA lentivirus (Fig. 3F), suggesting the positive relationship between Nrf2 and p62. When treated with different concentrations of doxorubicin for 24 h, cell proliferation rates of MCF‐7/ADR with Nrf2 or p62 shRNA lentivirus were decreased more significantly than that of control siRNA (Fig. 3G).

### PA‐MSHA inhibited MCF‐7/ADR cells proliferation through Nrf2/p62 in vitro

**Figure 4:**
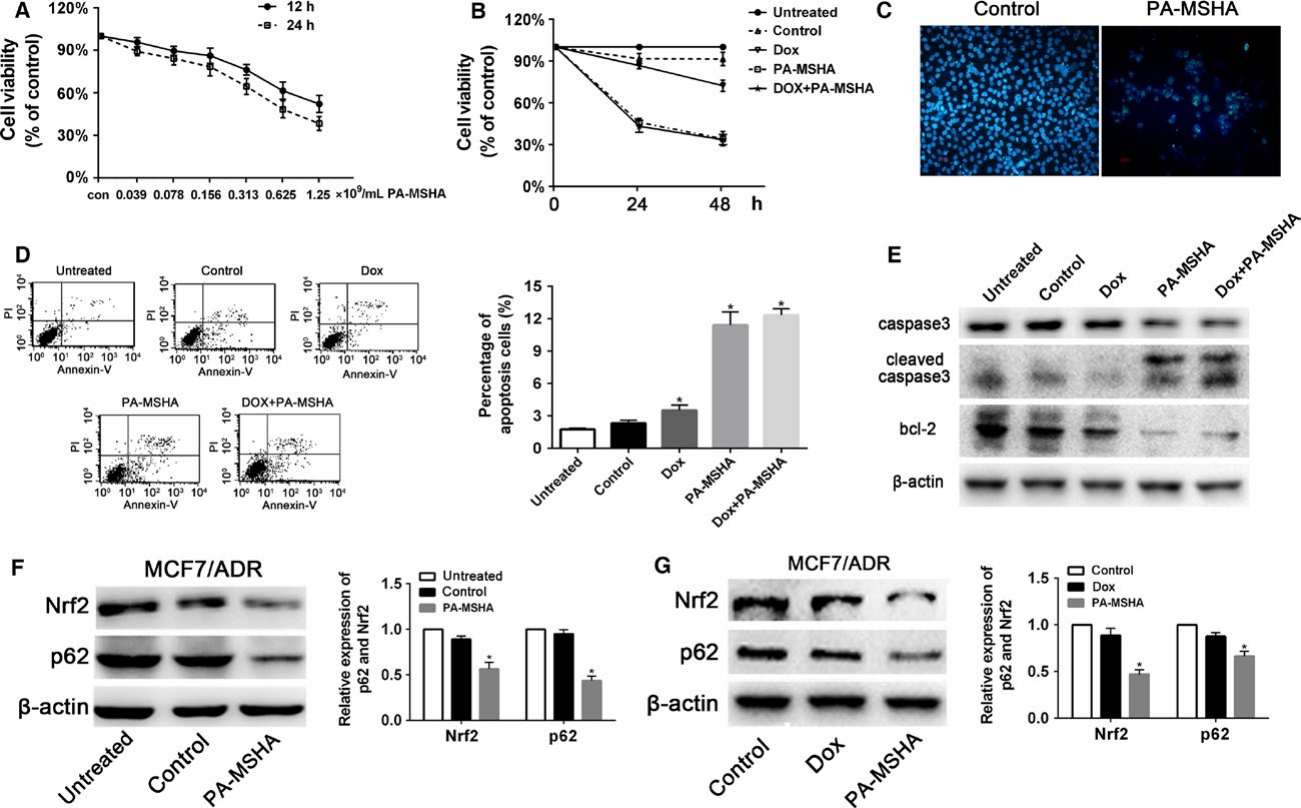
PA‐MSHA inhibited MCF‐7/ADR cells proliferation through Nrf2/p62 in vitro. (A) MCF‐7/ADR cells were treated with various concentrations of PA‐MSHA for 12 h and 24 h, and cell viability was determined by the CCK8 assay. (B) The inhibitory effect of different drugs on MCF‐7/ADR cell proliferation. Cells were treated with PBS, doxorubicin (3 *μ*g/mL), PA‐MSHA (0.848 × 10^9^cells/mL), and doxorubicin (3 *μ*g/mL)+PA‐MSHA (0.848 × 10^9^cells/mL) for 48 h, and cell viability was determined by the CCK8 assay. (C) The effect of different drugs on MCF‐7/ADR cell apoptosis. Nucleus of MCF‐7/ADR cells were stained by Hoechst 33258. Cells were treated with PBS or PA‐MSHA (0.848 × 10^9^cells/mL) for 48 h. (D) MCF‐7/ADR cells were treated with PBS, doxorubicin (3 *μ*g/mL), PA‐MSHA (0.848 × 10^9^cells/mL), and doxorubicin (3 *μ*g/mL)+PA‐MSHA (0.848 × 10^9^cells/mL) for 48 h, and the apoptotic fraction of cells was detected by Annexin V staining/propidium iodide staining. (E) MCF7/ADR cells were treated with PBS, doxorubicin (3 *μ*g/mL), PA‐MSHA (0.848 × 10^9^cells/mL), and doxorubicin (3 *μ*g/mL)+PA‐MSHA (0.848 × 10^9^cells/mL) for 48 h, then the protein levels of caspase 3, cleaved‐caspase 3, and bcl‐2 were detected by western blot. (F) MCF7/ADR cells were treated with PBS, PA‐MSHA (0.848 × 10^9^cells/mL) for 8 h, then the protein levels of Nrf2 and p62 were detected by western blot. (G) MCF7/ADR cells were treated with PBS, doxorubicin (3 *μ*g/mL), and PA‐MSHA (0.848 × 10^9^cells/mL) for 48 h, then the protein levels of Nrf2 and p62 were detected by western blot. PA‐MSHA, *Pseudomonas aeruginosa* mannose‐sensitive hemagglutinin; *P <0.05. PBS, phosphate‐buffered saline.

CCK8 assays showed that PA‐MSHA inhibited growth of MCF‐7/ADR. The 50% inhibition concentration (IC_50_) for PA‐MSHA in MCF‐7/ADR cells was 1.421 × 10^9^cells/mL at 12 h, while the IC_50_ was 0.848 × 10^9^cells/mL at 24 h (Fig. 4A). When treated with PA‐MSHA (0.848 × 10^9 ^cells/mL), the MCF‐7/ADR cell viability was lower than that treated with doxorubicin. However, compared with the group treated with PA‐MSHA alone, the percent of cell viability was not significantly decreased in the group treated with PA‐MSHA+doxorubicin (*P* > 0.05, Fig. 4B).

Treatment of MCF‐7/ADR with PA‐MSHA for 48 h resulted in morphological changes including nuclear condensation and nuclear fragmentation compared with control (Fig. 4C). This phenomenon was further confirmed by flow cytometric analysis. Compared with untreated (1.75 ± 0.12), control (2.32 ± 0.27), and doxorubicin (3.41 ± 0.33) groups, the percentage of apoptosis cells were higher in the group treated with PA‐MSHA (11.98 ± 0.28) or PA‐MSHA+doxorubicin (11.88 ± 0.24). However, there are no statistically significant differences of apoptosis cells between PA‐MSHA and PA‐MSHA+doxorubicin group (*P* > 0.05, Fig. 4D). Apoptosis‐associated caspase 3 activity was activated in PA‐MSHA and PA‐MSHA+doxorubicin group compared with untreated, control, and doxorubicin groups. The antiapoptotic protein bcl‐2 was reduced in PA‐MSHA or PA‐MSHA+doxorubicin group (Fig. 4E).

As the role of Nrf2/p62 in modulating the sensitivity of breast cancer cells to doxorubicin mentioned above, the effect of PA‐MSHA on Nrf2 and p62 was detected by western blot. After treated with PA‐MSHA, the protein levels of Nrf2 and p62 were significantly decreased in MCF‐7/ADR cells (Fig. 4F). However, the protein levels of Nrf2 and p62 did not change significantly in MCF‐7/ADR cells treated with Dox (Fig. 4G).

### PA‐MSHA inhibited MCF‐7/ADR xenograft tumor growth in vivo

**Figure 5:**
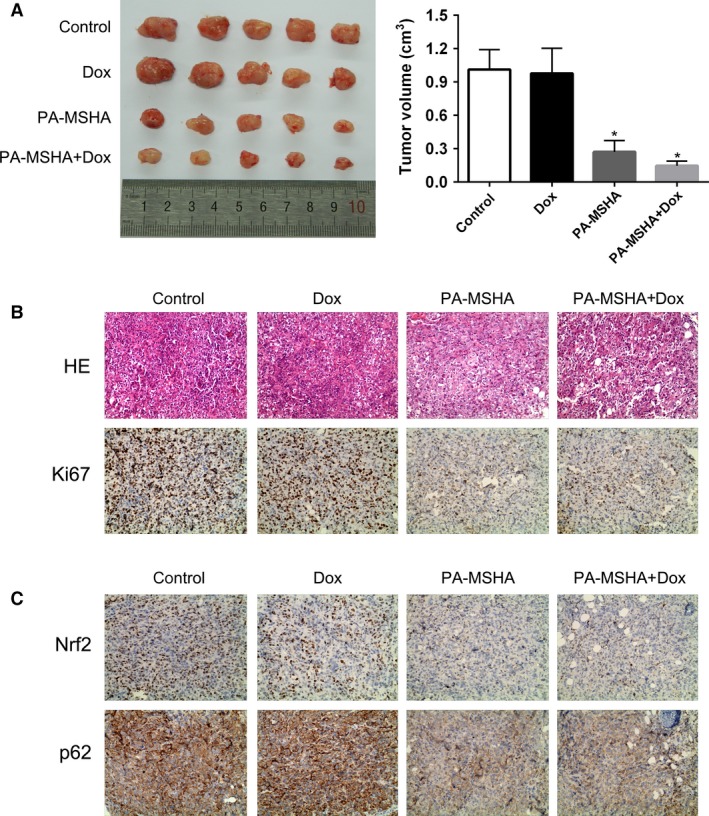
PA‐MSHA inhibited MCF‐7/ADR xenograft tumor growth in vivo. (A) Tumor volume measured at the indicated time after MCF‐7/ADR cells were implanted into the mammary fat pad of mice. (B) Histopathology of xenograft tumors stained with H&E and anti‐Ki67 antibody. (C) Histopathology of xenograft tumors. Tumor sections stained with anti‐Nrf2 and anti‐p62. Original magnification 400×, *P < 0.05. PA‐MSHA, *Pseudomonas aeruginosa* mannose‐sensitive hemagglutinin.

MCF‐7/ADR cells suspensions were injected into nude mice to form xenograft tumors. PA‐MSHA or PA‐MSHA+doxorubicin treatment led to significant tumor suppression in vivo, while PBS or Dox treatment did not reduce the volume of tumor xenografts (Fig. 5A). The xenograft tumors were analyzed by IHC with proliferation marker Ki67 staining. The data showed that the proliferative activity was lower in PA‐MSHA and PA‐MSHA+doxorubicin groups than in PBS and doxorubicin group (Fig. 5B). The MCF‐7/ADR xenograft tumors were also analyzed by IHC with Nrf2 and p62 staining. Compared with PBS and doxorubicin group, Nrf2 and p62 expression were decreased in PA‐MSHA group (Fig. 5C).

## Discussion

Several novel findings have reported the relationship between p62 and Nrf2. First, overexpression of p62 was able to disrupt the association between Nrf2 and Keap1 (kelch‐like ECH‐associated protein 1, a negative regulator of Nrf2), and then lead to activation of Nrf2 25. Second, p62 gene contain an ARE (antioxidant response element) in its promoter, which is a specific Nrf2‐binding site by which Nrf2 induces p62 transcription 12. Moreover, Nrf2 and p62 were upregulated in some cancers such as liver cancer 26, gliomas 27, ovarian cancer 9, and breast cancer 28. In this study, we confirmed that Nrf2 and p62 were overexpressed and there was a positive correlation between them in breast cancer tissues and cells.

Doxorubicin is a potent anthracycline chemotherapeutic agent and is widely used for the treatment of breast cancer. However, the clinical application of doxorubicin is limited mainly by drug resistance and dose‐dependent toxicity in normal organs. Recently, emerging data have revealed that constitutive activation of Nrf2 is thought to be responsible for acquired doxorubicin resistance 13, 29, 30, 31, 32. In addition, downregulation of p62 resulted in increased doxorubicin‐induced apoptosis in hepatocellular carcinoma 33. But whether p62 was involved in doxorubicin resistance in breast cancer has not been reported yet. In our study, we found that the expressions of Nrf2 or p62 in MCF‐7/ADR cells are higher than that in MCF‐7 cells. Moreover, MCF‐7/ADR cells were more resistant to doxorubicin than MCF‐7 cells. Silencing of Nrf2 or p62, respectively, decreased cell viability of MCF‐7/ADR cells treated with doxorubicin compared with the control group. When MCF‐7/ADR cells treated with doxorubicin, we found the expression of Nrf2 and p62 were increased significantly. These findings demonstrated upregulation of Nrf2 and p62 play an important role in breast cancer doxorubicin resistance.

The most frequent mechanisms in doxorubicin resistance are involved in decreased intracellular concentration of the drug due to the expression of multidrug resistance (MDR) proteins and many membrane efflux pumps such as p‐gp, increased drug metabolism enzymes such as glutathione S‐transferase GST, reduced concentration and activity of TOP2A, and failure of the cellular apoptotic pathways 34. Thus, targeting MDR is a promising approach to reducing the need for additional chemotherapeutic drugs. Nrf2 could upregulate ATP‐binding cassette transporters like MRP3, MRP4, or MRP5, facilitating the efflux of anticancer drugs, Nrf2‐inducible metabolizing enzymes and target genes contribute to the detoxification of anticancer drugs and thereby to chemoresistance. Moreover, Nrf2‐induced expression of survival genes like BCL2 contributes to reduced apoptotic responses to anticancer drugs 35. In human ovarian cancer cells, p62 efficiently regulated the Keap1–Nrf2–ARE system to mediate cisplatin resistance, avoiding oxidative stress‐induced apoptosis 9. Therefore, it is feasible to use Nrf2 and p62 inhibitors to overcome doxorubicin resistance.

Interestingly, in present study, we found that Nrf2 and p62 were both decreased in PA‐MSHA group. The PA‐MSHA strain is a peritrichous *P. aeruginosa* strain with MSHA fimbriae established by Professor Xi‐ya Mu. PA‐MSHA possesses cytotoxic qualities due to the addition of MSHA, which has been shown to have anticarcinogenic activity. PA‐MSHA could efficiently inhibit proliferation and induce apoptosis, which is associated with the inactivation of EGFR signaling pathway 16, 17, 36. PA‐MSHA was found to induce endoplasmic reticulum (ER) stress in breast cancer cell lines through the IRE1 signaling pathway. Inhibiting autophagy potentiated the cytotoxic effect of PA‐MSHA while treating MDA‐MB‐231 and MDA‐MB‐468 breast cancer cell lines 37. In contrast, MCF‐7 cell line was relatively resistant to PA‐MSHA 16, 38, which were consistent with our results (Fig. S1). However, whether PA‐MSHA exerts the cytotoxic effect on doxorubicin resistance breast cancer cells has not been reported so far. In our study, we found PA‐MSHA could inhibit the growth and induce the apoptosis of MCF‐7/ADR cells in vitro and vivo.

Although upregulation of Nrf2 and p62 and their roles in chemoresistance have been reported in other cancer types, in our present article, we focus on the growth inhibition of doxorubicin‐resistant MCF‐7/ADR by PA‐MSHA via Nrf2/p62. In Figure S2 we found that PA‐MSHA significantly inhibited the proliferation of MCF‐7/ADR and the expression levels of Nrf2 and p62 in the presence of doxorubicin, these effects can be blocked by tBHQ. Taken together, we showed Nrf2 and p62 were both overexpressed in breast cancer cells and tissues. Stable knockdown of Nrf2 or p62 significantly sensitized breast cancer cells to doxorubicin. PA‐MSHA inhibited growth and induced apoptosis of MCF‐7/ADR cells but not MCF‐7 cells. In addition, PA‐MSHA could downregulate Nrf2 and p62 in vitro and in MCF‐7/ADR xenograft model. It suggested that PA‐MSHA inhibits the growth of doxorubicin‐resistant MCF‐7/ADR cells by downregulating Nrf2/p62. However, the underlying mechanism of the suppression of Nrf2/p62 pathway by PA‐MSHA is still unknown, which would be investigated in our further study.

## Conflict of Interest

The authors declare that they have no competing interests.

## Supporting information


**Figure S1.** The inhibitory effect of PA‐MSHA (0.848 × 10^9^cells/mL) on MCF‐7/ADR and MCF‐7 cell proliferation for 12 h and 24 h. The cell viability was determined by the CCK8 assay.

**Figure S2.** (A) The protein levels of Nrf2 and p62 were determined by western blot in MCF‐7/ADR cells after pretreated with tBHQ and/or PA‐MSHA. (B) The tBHQ/PA‐MSHA pretreated MCF‐7/ADR cells were treated with the indicated doses of doxorubicin for 24 h, followed by the CCK8 assay.

**Figure S3.** (A) Western blot analysis of Nrf2 and p62 protein levels in MCF‐7, T47D, BT549, and MCF‐7/ADR breast cancer cells. (B) MCF‐7, T47D, BT549, and MCF‐7/ADR cells were treated with the indicated doses of doxorubicin for 24 h, followed by the CCK8 assay.

**Figure S4**. The inhibitory effect of different concentrations of drugs on MCF‐7/ADR cell proliferation. Cells were treated with doxorubicin (3 *μ*g/mL), PA‐MSHA (0.284 × 10^9^cells/mL), PA‐MSHA (0.503 × 10^9^cells/mL), PA‐MSHA (0.848 × 10^9^cells/mL), doxorubicin+PA‐MSHA (0.284 × 10^9^cells/mL), doxorubicin+PA‐MSHA (0.503 × 10^9^cells/mL), and doxorubicin+PA‐MSHA (0.848 × 10^9^cells/mL) for 48 h, and cell viability was determined by the CCK8 assay.

**Figure S5**. The protein levels of Nrf2 and p62 were determined by western blot in MCF‐7/ADR cells after pretreated with PA‐MSHA or/and doxorubicin.
