# Supplementary figures and images for: PA‐MSHA inhibits the growth of doxorubicin‐resistant MCF‐7/ADR human breast cancer cells by downregulating Nrf2/p62
Source: Cancer Med. 2016 Oct 18;5(12):3520–31. doi: 10.1002/cam4.938 (PMC5224842; doi:10.1002/cam4.938)

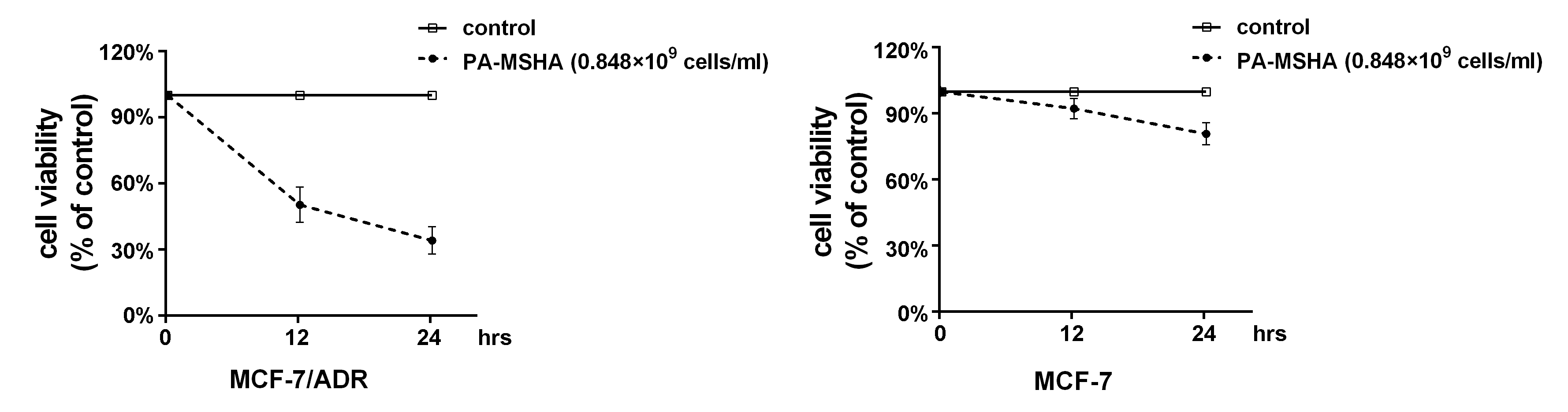

Supplement: Supplementary file 1 — Figure S1. The inhibitory effect of PA‐MSHA (0.848 × 10^9^cells/mL) on MCF‐7/ADR and MCF‐7 cell proliferation for 12 h and 24 h. The cell viability was determined by the CCK8 assay. [file CAM4-5-3520-s001.tif]

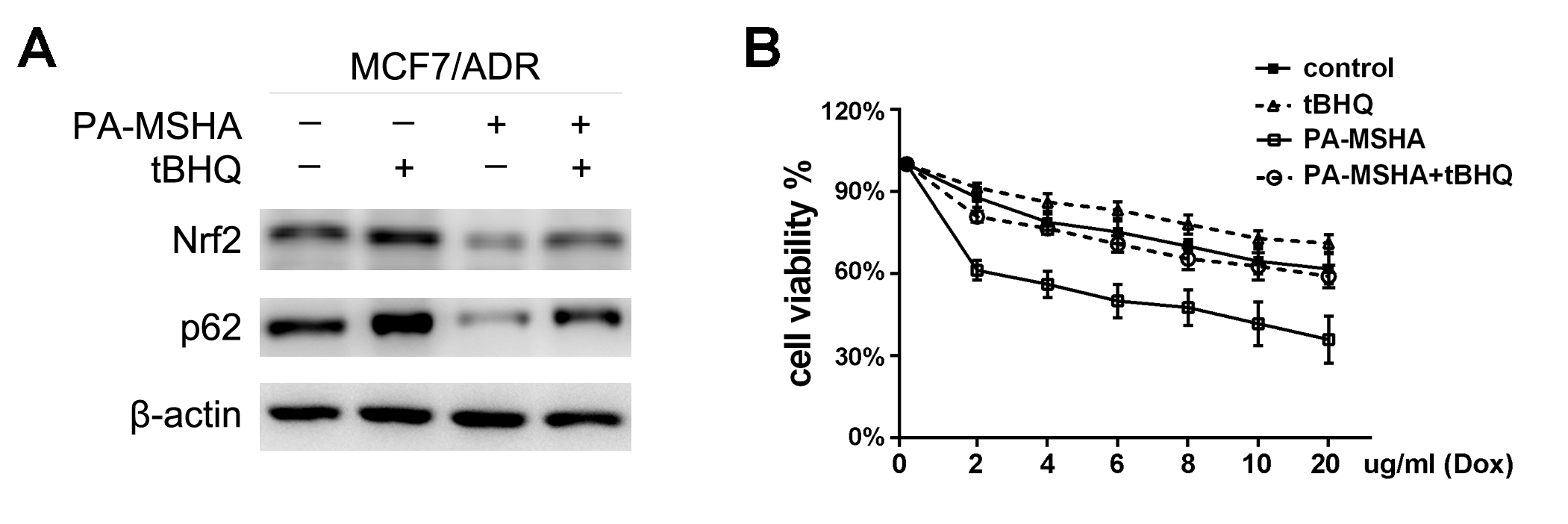

Supplement: Supplementary file 2 — Figure S2. (A) The protein levels of Nrf2 and p62 were determined by western blot in MCF‐7/ADR cells after pretreated with tBHQ and/or PA‐MSHA. (B) The tBHQ/PA‐MSHA pretreated MCF‐7/ADR cells were treated with the indicated doses of doxorubicin for 24 h, followed by the CCK8 assay. [file CAM4-5-3520-s002.tif]

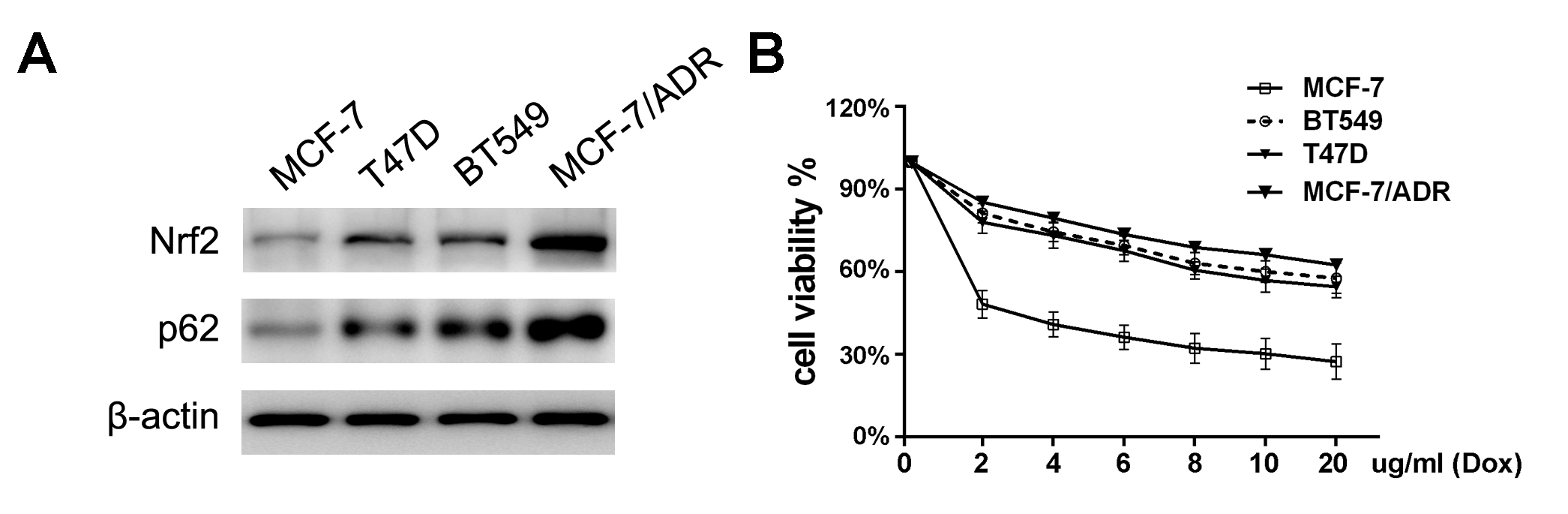

Supplement: Supplementary file 3 — Figure S3. (A) Western blot analysis of Nrf2 and p62 protein levels in MCF‐7, T47D, BT549, and MCF‐7/ADR breast cancer cells. (B) MCF‐7, T47D, BT549, and MCF‐7/ADR cells were treated with the indicated doses of doxorubicin for 24 h, followed by the CCK8 assay. [file CAM4-5-3520-s003.tif]

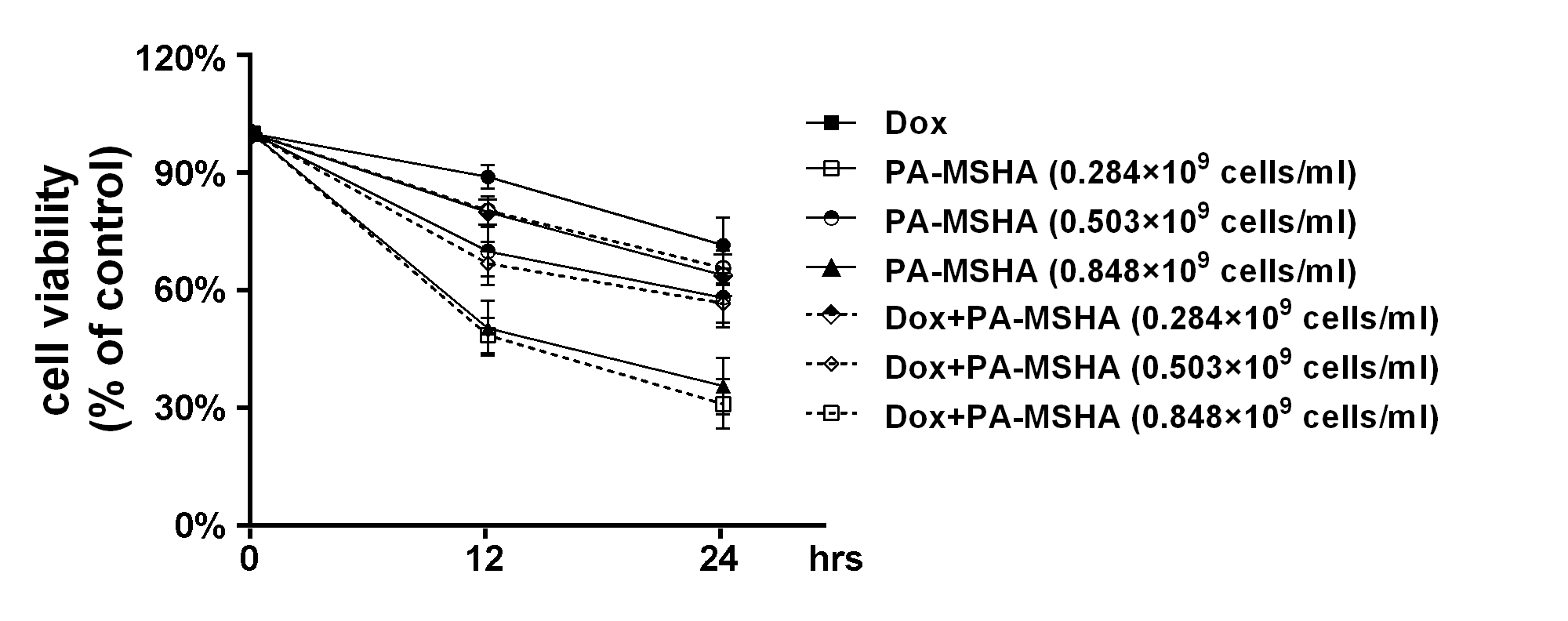

Supplement: Supplementary file 4 — Figure S4. The inhibitory effect of different concentrations of drugs on MCF‐7/ADR cell proliferation. Cells were treated with doxorubicin (3 μg/mL), PA‐MSHA (0.284 × 10^9^cells/mL), PA‐MSHA (0.503 × 10^9^cells/mL), PA‐MSHA (0.848 × 10^9^cells/mL), doxorubicin+PA‐MSHA (0.284 × 10^9^cells/mL), doxorubicin+PA‐MSHA (0.503 × 10^9^cells/mL), and doxorubicin+PA‐MSHA (0.848 × 10^9^cells/mL) for 48 h, and cell viability was determined by the CCK8 assay. [file CAM4-5-3520-s004.tif]

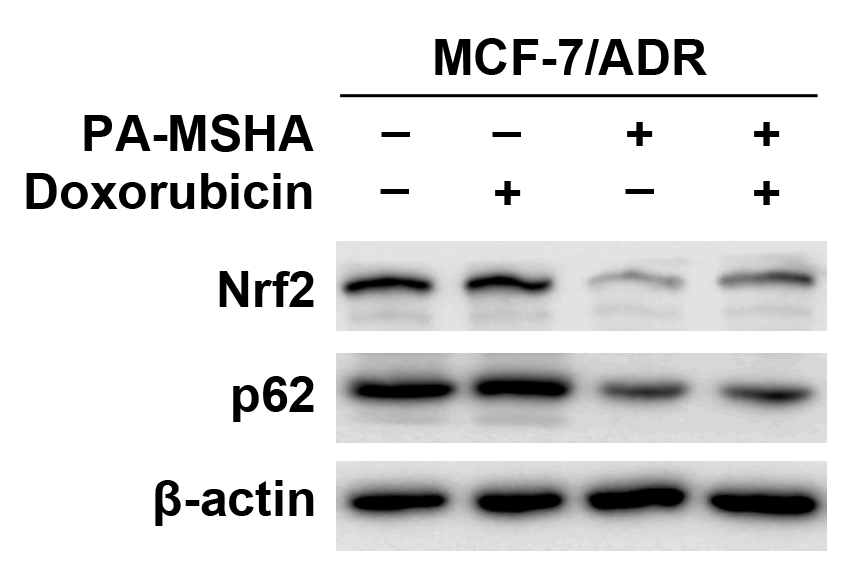

Supplement: Supplementary file 5 — Figure S5. The protein levels of Nrf2 and p62 were determined by western blot in MCF‐7/ADR cells after pretreated with PA‐MSHA or/and doxorubicin. [file CAM4-5-3520-s005.tif]
